# Mechanistic insight into sevoflurane-associated developmental neurotoxicity

**DOI:** 10.1007/s10565-021-09677-y

**Published:** 2021-11-12

**Authors:** Mingyang Sun, Zhongcong Xie, Jiaqiang Zhang, Yufang Leng

**Affiliations:** 1 The First School of Clinical Medicine, Lanzhou University, Lanzhou, Gansu People’s Republic of China 730000; 2 Department of Anesthesiology and Perioperative Medicine, Center for Clinical Single Cell Biomedicine, Henan Provincial People’s Hospital, People’s Hospital of Zhengzhou University, Zhengzhou, Henan People’s Republic of China 450003; 3 Department of Anesthesia, Critical Care and Pain Medicine, Massachusetts General Hospital and Harvard Medical School, Boston, MA USA; 4 Day Surgery Center, The First Hospital of Lanzhou University, Lanzhou, Gansu People’s Republic of China 730000

**Keywords:** Sevoflurane, Young brain, Neural cell damage, Neural circuit, Tau phosphorylation, Neuroendocrine

## Abstract

With the development of technology, more infants receive general anesthesia for surgery, other interventions, or clinical examination at an early stage after birth. However, whether general anesthetics can affect the function and structure of the developing infant brain remains an important, complex, and controversial issue. Sevoflurane is the most-used anesthetic in infants, but this drug is potentially neurotoxic. Short or single exposure to sevoflurane has a weak effect on cognitive function, while long or repeated exposure to general anesthetics may cause cognitive dysfunction. This review focuses on the mechanisms by which sevoflurane exposure during development may induce long-lasting undesirable effects on the brain. We review neural cell death, neural cell damage, impaired assembly and plasticity of neural circuits, tau phosphorylation, and neuroendocrine effects as important mechanisms for sevoflurane-induced developmental neurotoxicity. More advanced technologies and methods should be applied to determine the underlying mechanism(s) and guide prevention and treatment of sevoflurane-induced neurotoxicity.

## Highlights

1. We discuss the mechanisms underlying sevoflurane-induced developmental neurotoxicity from five perspectives: neural cell death, neural cell damage, assembly and plasticity of neural circuits, tau phosphorylation, and neuroendocrine effects. 2. Tau phosphorylation, IL-6, and mitochondrial dysfunction could interact with each other to cause a nerve damage loop. 3. miRNAs and lncRNAs are associated with sevoflurane-induced neurotoxicity.

## Introduction

With the progress of medical technology, greater numbers of infants receive surgery, interventions, or examination under general anesthesia at an early stage after birth. Consequently, the neurotoxicity of general anesthesia is receiving increasing attention. The US Food and Drug Administration released a warning of drug safety in December 2016, which stated that 11 commonly used sedative and anesthetic medications had potential neurotoxic effects in pregnant women during the third trimester and children under the 3 years of age (2016).This warning indicates the need for studies to delineate any potential adverse neurodevelopmental consequences on children.

Sevoflurane is an agentia for inhalational induction due to its low blood-gas solubility and rapid onset, particularly in pediatric anesthesia. Yet, repeated or prolonged exposure to sevoflurane is neurotoxic in the developing brain in animal studies. For example, in mice, maternal anesthesia with sevoflurane induces social interaction deficits in the offspring (Chen et al., 2021). Monkeys exposed to sevoflurane anesthesia in infancy have increased anxiety-related behaviors during adolescence, (Raper et al., 2015), and early repeated sevoflurane anesthesia in monkey infancy results in an anxious phenotype which persists over time (Raper et al., 2018). In mice, neonatal exposure to sevoflurane anesthesia may raise the risk of cognitive dysfunction in adults (Dai et al., 2020), and repeated neonatal sevoflurane exposure induces attention-deficit/hyperactivity-disorder-like impulsive behavior in later adulthood (Xie et al., 2020).

Findings in humans are less conclusive. Children under 3 years of age who underwent surgery were 60% more likely to be subsequently diagnosed with developmental and behavioral disorders than children accepted no surgery (DiMaggio et al., 2011), and anesthesia was considered to be a potential independent risk factor. Most recently, both of the General Anesthesia vs. Spinal (GAS) Anesthesia and Pediatric Anesthesia and Neurodevelopment Assessment (PANDA)studies involved formal neurodevelopmental testing, showed that there is no correlation between single and transient general anesthesia and poor neurodevelopmental outcome. (Davidson et al., 2016; Sun et al., 2016). However, in the Mayo Anesthesia Tolerability in Kids study, there were subtle declines in fine-motor coordination and processing speed that might impede learning after sevoflurane exposure, and children one or more times exposed to sevoflurane had difficulty with reading (Warner et al., 2018). Furthermore, a comparison of 46 neurodevelopmental outcomes in 13,433 children showed that multiple exposures to sevoflurane are associated with an increased risk of poor motor function, lower hand dexterity, and lower social scores (Walkden et al., 2020).

**Fig. 1:**
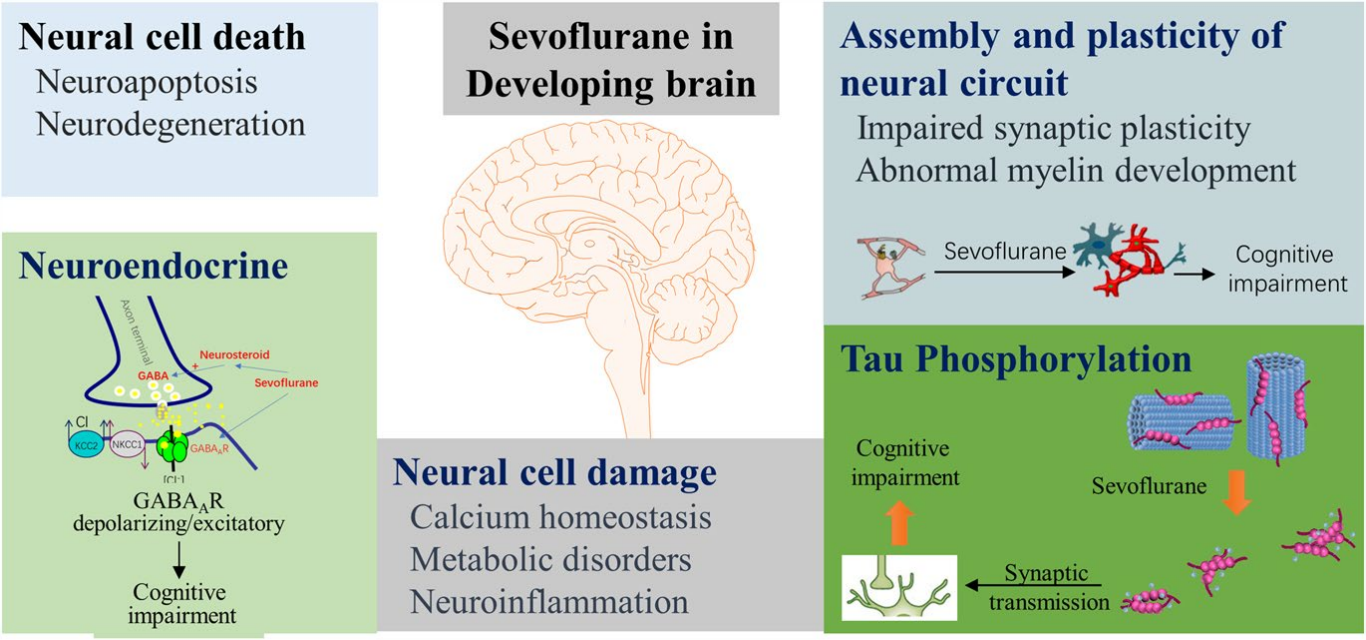
The mechanisms of sevoflurane may alter neurodevelopment from five aspects: neural cell death, neural cell damage, assembly and plasticity of the neural circuit, tau phosphorylation, and neuroendocrine effects

Together, existing findings from both animal studies and clinical studies raise concern about the potential neurotoxic effect of sevoflurane in children, particularly for any interference with critical processes in brain development. In this review, we discuss the mechanisms by which sevoflurane may alter neurodevelopment from five aspects: neural cell death, neural cell damage, assembly and plasticity of the neural circuit, tau phosphorylation, and neuroendocrine effects (Fig. 1).

## Neural cell death

### Neuroapoptosis is the main pathway leading to neural cell death

Most pre-clinical studies of sevoflurane focus on apoptosis as the leading cause of neurotoxicity. During development, this type of programmed cell death is normal, but prolonged exposure to sevoflurane in young animals could lead to the rate of neuroapoptosis increasing 50-fold (McCann and de Graaff, 2017). Sevoflurane exposure causes millions of otherwise healthy and functional neurons to commit to apoptotic-programmed cell death (Dikranian et al., 2001). Indeed, this exposure can cause neuroapoptosis through several different molecular mechanisms, and it is not yet clear which mechanism is preferred. Two major signaling pathways can promote apoptosis: the intrinsic and the extrinsic pathways (Yon et al., 2005).

The intrinsic pathway is initiated in response to signals from within the cell, resulting in a decreasing anti-apoptotic (such as BCL-2, MCL1, BCL-XL) and pro-apoptotic (BAX/BAK) ratio, which induces mitochondrial outer membrane permeabilization (MOMP). MOMP promoted cytochrome C release from the mitochondria and activated caspase-9 cleavage (Green and Llambi, 2015; Liu et al., 2020; Yon et al., 2005). Phosphorylation of antiapoptotic MCL1 can be activated by stress and mitotic kinases such as AMP-activated protein kinase (MAPK), p38 MAPK, casein kinase II, Jun amino-terminal kinase (JNK) (Wertz et al., 2011), and growth factor and phosphoinositide 3-kinase (PI3K)-AKT-glycogen synthase kinase (GSK) 3 signaling (Maurer et al., 2006). BH3-only proteins (sensitizers or derepressors) promote MOMP proteins, BCL-2. JNK, extracellular signal-regulated kinase 1 and 2 (ERK1/2) by antagonizing antiapoptotic and p53 are involved in apoptosis, and their activation can promote MOMP and induce intrinsic apoptosis (Green and Llambi, 2015). Neonatal sevoflurane exposure in rodents profoundly decreases the histone acetyltransferase activity of cyclic adenosine monophosphate (cAMP) response element-binding (CREB) protein in the hippocampus (Dong et al., 2020a). CREB-binding protein acetylation is implicated in learning (Barrett et al., 2011; Maddox et al., 2013), and CREB pathway inactivation may downregulate the transcription of anti-apoptotic genes and increase the levels of pro-apoptotic factors (Lee et al., 2013), in turn, decreasing the anti-apoptotic (BCL-2, MCL1, BCL-XL)/pro-apoptotic (BAX/BAK) ratio, promoting MOMP, and inducing intrinsic apoptosis.

Sevoflurane induces neuroapoptosis in the developing brains of young rats via the brain-derived neurotrophic factor (BDNF)-modulated apoptotic cascade (Hu et al., 2019; Yu et al., 2020b), which also results in a decreasing anti-apoptotic/pro-apoptotic ratio, promoting MOMP, and inducing intrinsic apoptosis. Sevoflurane exposure in the developing brain could induce neuroapoptosis by activating the JNK/c-JUN/AP-1 signaling pathway (Bi et al., 2018), activation/phosphorylation of ERK1/2 via β-arrestin 1 and 2/metabotropic glutamate receptor 7 (Wang et al., 2016a, b), activation/phosphorylation of the ERK1/2-NRF2/BACH1 signaling pathway (Yang et al., 2020a), activation/phosphorylation of ERK1/2-MAPK signaling (Wang et al., 2016a, 2013b), and activation/phosphorylation of the PI3K/AKT signaling pathway (Li et al., 2017a), which can also decrease the anti-apoptotic/pro-apoptotic ratio, promote MOMP, induce intrinsic apoptosis, and cause cognitive decline in adolescence.

**Fig. 2:**
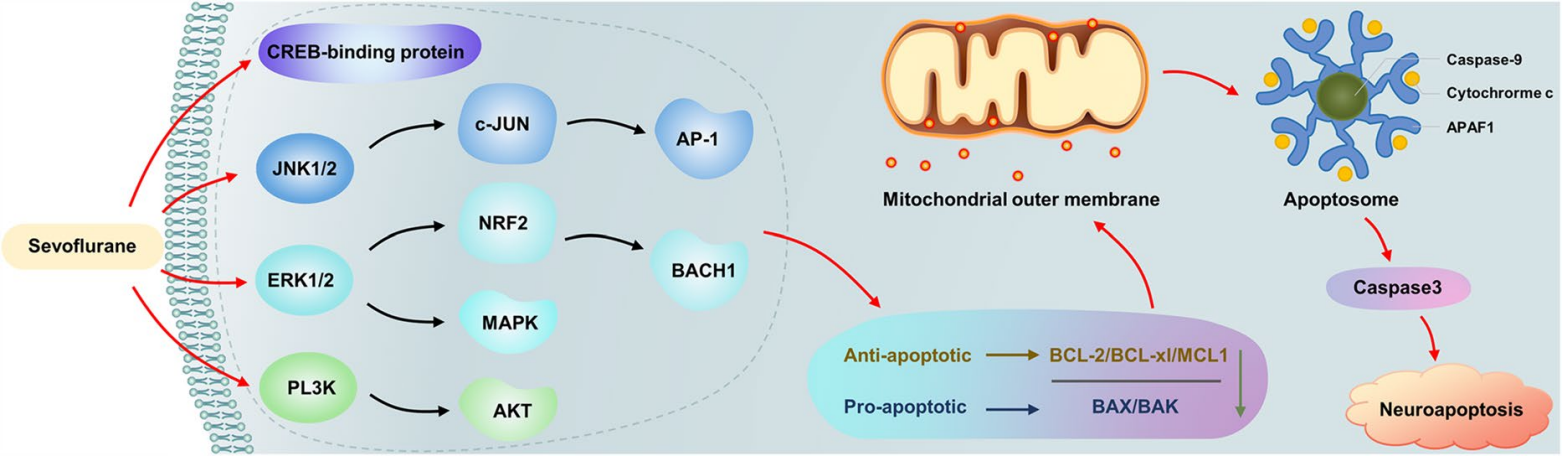
Sevoflurane exposure in the developing brain could induce neuroapoptosis via the JNK/c-JUN/AP-1 axis, ERK1/2 MAPK axis, ERK1/2-NRF2/BACH1axis, PI3K/AKT axis, and CREB inactivation, which may downregulate the transcription of anti-apoptotic genes (BCL-2, BCL-XL, MCL1) and increase the levels of pro-apoptotic factors (BAX/BAK). This induces mitochondrial outer membrane permeabilization, promoting release of cytochrome C from the mitochondria, activating caspase-9 cleavage and informed apoptosome, resulting in caspase 3 activation and neuroapoptosis

Repeated sevoflurane anesthesia treatment in neonatal rats also increases the density of NeuN + /caspase-3 + cells, which in the hippocampal dentate gyrus, induces caspase-3 activation, and increases BAX levels. It also reduces levels of BCL-2 in adolescent rats, which decreases the ratio of BCL-2/BAX, promotes MOMP, induces intrinsic apoptosis, and causes cognitive decline in adolescence. In clinical studies, sevoflurane exposure increases the mRNA expression levels of caspase-3, superoxide dismutase 1, and glutathione peroxidase gene 1 in neural stem cells (NSCs) in postoperative blood samples and reduces cell density and cell viability of NSCs in postoperative serum in children less than 3 years old (Zhou et al., 2018). Long noncoding RNAs (lncRNA) expression profiles in the developing mouse hippocampus indicate that sevoflurane exposure upregulates lncRNAs, likely induces over-expression of BCL2L11 and BAX, but decreases BCL-2, which eventually promotes mitochondria-mediated apoptosis (Chen et al., 2016). Sevoflurane induces neuronal apoptosis in the immature brain mostly by initiating the intrinsic pathway. The endogenous apoptosis pathway could be targeted to treat neurotoxicity caused by sevoflurane in the developing brain (Fig. 2).

The extrinsic pathway can be activated via tumor necrosis factor (TNF) receptors, which increase FAS (also known as CD95, which is a death receptor) protein and activate caspase-8. Cell death induced by death receptors is generally crucial to immune system function and homeostasis (Green and Llambi, 2015). Bid is activated by caspase-8-mediated cleavage, and Bid in turn promotes MOMP by activating BAX and BAK. Sevoflurane significantly increases the expression of FAS protein in young mice (Song et al., 2015), which activates caspase-8 and induces neuroapoptosis. However, sometimes sevoflurane-induced apoptosis may be independent of death receptor signaling (Loop et al., 2005). Furthermore, the extrinsic and intrinsic pathways may at times interact with each other to cause apoptosis.

Sevoflurane also induces neuroapoptosis through modulation of reactive oxygen species (ROS) production (Jin et al., 2013), proopiomelanocortin (Wei et al., 2019b), and β-amyloid protein (Lu et al., 2010), shifting the pentose phosphate pathway to the glycolytic pathway (Liu et al., 2019a). This also affects the receptor-interacting protein (RIPK)1/RIPK3 signaling pathway (Xu et al., 2021) and the PERK-eIF2a ATF4- CHOP axis of the endoplasmic reticulum (ER) stress signaling pathway (Liu et al., 2017a). The increasing intracellular ROS results in DNA damage (Piao et al., 2020), and disruption of intracellular calcium homeostasis is also observed (Yang and Wei, 2017). Neuroapoptosis is an important mechanism of developmental neurotoxicity induced by sevoflurane. Inhibiting the neuroapoptosis pathway could be a potential targeted intervention for neurotoxicity induced by sevoflurane in the developing brain.

### Neurodegeneration is the pathological basis of many cognitive disorders

**Fig. 3:**
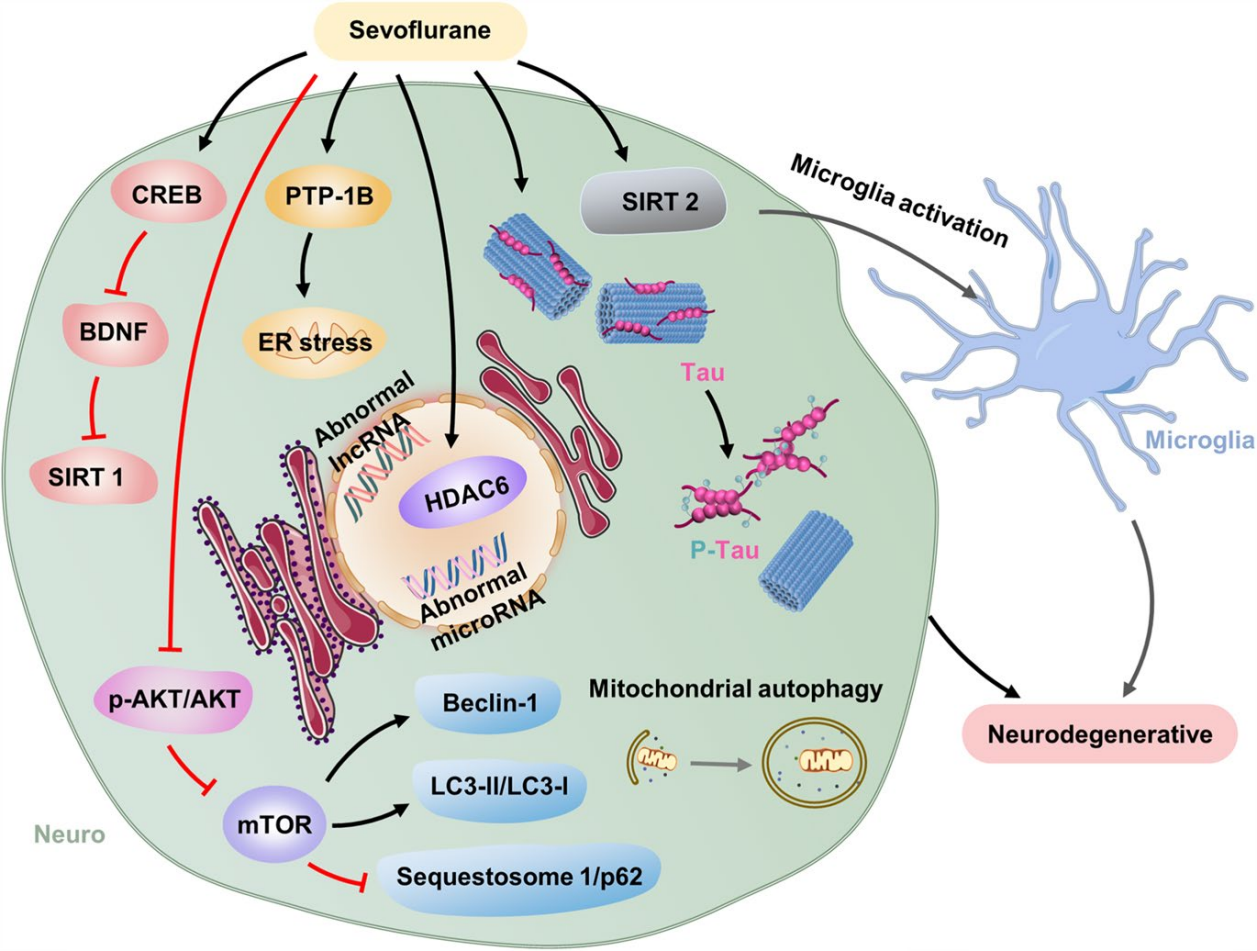
Sevoflurane could induce neurodegenerative in the developing brain through multiple signaling pathways such as PTP-1B/ER axis, CREB/BDNF/SIRT1 axis, SIRT 2/microglia activation, and HDAC6 overexpression. Sevoflurane exposure suppressed p-AKT/AKT and mTOR, increasing the level of Beclin-1 and LC3-II/LC3-I and decreasing the levels of sequestosome 1 and p62, which induce activation of autophagy. Autophagy could induce neurodegenerative, which involved in sevoflurane-induced neurodegenerative

Neurodegeneration underlies many cognitive disorders. In some studies, sevoflurane exposure is reported to cause neurodegenerative changes. Damage from these changes may be more widespread than initially assumed, and understanding the extent of this damage is crucial for identifying the mechanisms of and treatments for anesthesia-related neurotoxicity (Burks et al., 2020). In the pathogenesis of neurodegenerative disorders, ER stress plays a critical role (Hetz and Saxena, 2017). Sevoflurane exposure for the developing brain increases protein tyrosine phosphatase 1B, located in the ER, and triggers ER stress that leads to neurodegenerative changes (Liu et al., 2019b, 2017a; Zhu et al., 2017). Sevoflurane also induces neurodegeneration through restoring cAMP and activating the cAMP/CREB signaling pathway in the developing hippocampus (Chen et al., 2020; Huang et al., 2021). Abnormal cAMP signal is an important factor in neurodegenerative changes. The methyl-cytosine-phosphate-guanine-binding protein 2/CREB signaling pathway downregulates BDNF, which inhibits the level of sirtuin 1(SIRT1) (Tang et al., 2020). Abnormal reduction of SIRT1 is also associated with neurodegenerative diseases. Sevoflurane significantly upregulates SIRT 2 in the neonatal rat hippocampus, which promotes pro-inflammatory/M1-related markers in microglia and activated microglia (Wu et al., 2020c). Microglia activation also has been implicated in neurodegenerative disease (Yeh et al., 2017). Multiple exposures to sevoflurane enhance histone deacetylase 6 (HDAC6) expression and activity in the developing hippocampus (Li et al., 2019a), and HDAC6 overexpression plays a very important role in neurodegenerative changes. A 3% sevoflurane exposure in neonatal induces abnormal lncRNA and microRNA (miRNA) expression profiles. The dysregulated lncRNAs/mRNAs were able to formulate wide molecular networks that might contribute to neurodegenerative signaling pathways, resulting in impaired long-term memory (Jiang et al., 2021b). Iron is essential for normal neuronal function; however, excess iron is implicated in several neurodegenerative diseases. Sevoflurane exposure disturbs iron homeostasis and causes iron overload in the hippocampus, which contributes to neurodegenerative diseases (Wu et al., 2020a). Protein aggregates and mitochondria dysfunction represent key pathological hallmarks shared by most neurodegenerative diseases. Autophagic flux, the difference between autophagosome formation and cargo clearance by lysosomes, is concerned of neurodegenerative diseases development (Budini et al., 2017; Heras-Sandoval et al., 2014; Klionsky et al., 2016; Ravikumar et al., 2005). LC3, the most widely monitored autophagy-ER-related protein, is commonly used as a marker of autophagy. The degradation of p62 in autolysosomes suggested as an indicator of autophagy activation (Klionsky et al., 2016). Sevoflurane increases the levels of microtubule-associated protein light chain 3II protein (LC3-II), beclin-1, and the ratio of LC3-II/LC3-I and decreases the levels of sequestosome 1 and p62 (Wei et al., 2019a; Xu et al., 2018) via suppression of phospho-protein kinase B/protein kinase B (p-AKT/AKT) and mammalian target of rapamycin (mTOR) (Li et al., 2017b) in the developing brain, inducing mitochondrial dysfunction and neurodegeneration. Repeated exposure of neonatal mice to 3% sevoflurane induces tau protein phosphorylation (Yu et al., 2020a), which leads to tau accumulation and the formation of neurofibrillary tangles, a hallmark pathology in neurodegenerative brains, suggesting that tau plays a critical role in neurodegeneration (Pirscoveanu et al., 2017; Yang and Wang, 2018). Neurodegenerative changes caused by neonatal sevoflurane exposure may be involved in a variety of pathways, but the exact mechanism is not clear, and further investigation is needed. (Fig. 3).

## Neural cell damage

### Calcium homeostasis deregulation can lead to neural cell damage

Calcium plays a vital role in human physiology, particularly in the central nervous system (CNS). Precise maintenance of Ca^2+^ levels is vital for normal cell function, and calcium homeostasis deregulation can lead to neuronal cell damage. ER is the main source of intracellular calcium release in neurons; it plays a very important role in the maintenance of intracellular calcium homeostasis (Wei and Xie, 2009). In the mitochondrial matrix, proper Ca^2+^ levels tightly regulate oxidative phosphorylation activity, which maintains the rate of adenosine triphosphate (ATP) production. However, if excess Ca^2+^ was taken up by mitochondria, derived from the increased cytosolic Ca2^+^ or excessive Ca^2+^ transfer from the ER, mitochondrial respiration can be impaired, which leads to enhanced production of ROS, impaired mitochondrial membrane permeabilization, and reduced ATP production, possibly with subsequent cell damage (Calvo-Rodriguez and Bacskai, 2021; Marchi et al., 2018; Mendes et al., 2005). Sevoflurane exposure induces a significant decrease of calcium concentrations in the ER via excessive IP3 receptors activation, the Ca^2+^ in the cytosol, and mitochondrial accompanied by a subsequent significant increase (Yang et al., 2008). Mitochondrial Ca^2+^ overload leads to mitochondrial respiration impairment, ROS activation, ATP reduction, and MOMP, which induce neural cell damage and apoptosis (Danese et al., 2017; Yang and Wei, 2017). Exposure to 2% sevoflurane at neonatal ages upregulates Ca^2+^-activated potassium channel type 2 (SK2s) in the CA1 region, which has persistent detrimental effects on long-term depression (LTD) and long-term potentiation (LTP) (Yu et al., 2018), and sevoflurane disrupts astrocyte Ca2^+^ homeostasis, which downregulates ezrin. The reduction of ezrin leads to astrocytic and neuronal dysfunction, which induces deficits in social behaviors of developing mice (Zhou et al., 2019). Imbalance of calcium homeostasis is an important mechanism of neurotoxicity induced by sevoflurane anesthesia, which is related to mitochondrial dysfunction and astrocytic and neuronal dysfunction. Maintaining intracellular calcium homeostasis may be an effective intervention for sevoflurane-induced developmental neurotoxicity.

### Metabolic disorders contribute to sevoflurane-induced neural cell damage

Sevoflurane exposure in the developing brain decreases the intermediates in the glucose metabolic pathway, including lactate, succinic acid levels significantly decrease, and the total creatine pool, including high-energy phosphocreatine and creatine are significantly reduced (Liu et al., 2015a). Total creatine pool depletion could increase the vulnerability of cellular to insufficiency of ATP synthesis, leading to cellular dysfunction (Liu et al., 2015a; Tsuji et al., 1995). In the neonatal brain, altered amino acid metabolism may also play a key role in sevoflurane-induced neurotoxicity. Prolonged and high concentration exposure to sevoflurane reduces levels of glutamine, glutamic acid, aspartic acid, and proline significantly. These amino acids are involved in the peptides, fatty acids, and synthesis of proteins, and reduction of their levels (Liu et al., 2015a) suggests neural cell damage and inhibition of neuronal growth in the developing brain. Additionally, prolonged sevoflurane anesthesia significantly reduces the levels of cadherin 1 (CDH1) in postnatal day 7 mice, which results in glucose metabolism switched from the pentose phosphate pathway to neuronal glycolysis. This conversion leads to an imbalance between the production of reactive oxygen species and decreased glutathione levels in the developing brain. The brain is more susceptible to oxidative stress, leading to cell damage. (Liu et al., 2019a). Sevoflurane-induced neuronal damage is also related to changes in lipid composition and content. Specific lipid changes can provide insight into the molecular mechanism of anesthesia-induced neurotoxicity, and may be a sensitive biomarker for anesthesia-induced neuronal damage, which can be used for early detection (Liu et al., 2015b).

Iron metabolism, folic acid metabolism, and other imbalances also can lead to nerve damage. The balance of brain iron metabolism is vital to the development of brain tissue. Especially in fetuses or infants, iron deficiency affects myelination and nerve tissue development, which plays a key role in cognitive function. Exposure to sevoflurane during pregnancy will reduce the expression of light chain ferritin, heavy chain ferritin, myelin basic protein tight junction protein ZO-1, claudin-5, occludin, and ferroportin-1 and increase the hippocampus and ferroportin-1 of offspring mice. Transferrin receptor 1 in the cortex, causing iron deficiency in the offspring’s brain and impaired myelin development (Zuo et al., 2021, 2020). Ferroptosis may also be involved in cognitive impairment caused by sevoflurane in the developing brain, which may be related to activation of N-methyl-D-aspartate receptor (NMDAR)-RASD1(Ras-related dexamethasone-induced 1) signaling (Wu et al., 2020a). Moreover, sevoflurane also leads to disrupted folate metabolism and subsequent defects in myelination in the developing brain (Zhang et al., 2019a). Metabolism is involved in all phases of cell growth, and metabolism disorders may lead to cell damage, which contributes to sevoflurane-induced neurotoxicity. However, there are only a few studies aimed at determining the role of metabolism in anesthesia developmental neurotoxicity.

### Neuroinflammation plays an important role in cognitive impairment

The primary characteristic of neuroinflammation is overexpression of proinflammatory factors from glial activation or immune cell infiltration. Neuroinflammation conventionally refers to the ability of the central and peripheral nervous system to generate innate immune responses during pathological events (Mendiola and Cardona, 2018). Mounting inflammatory responses to injury, astroglia, and microglia are considered the hallmark effector cells. Activated microglia are a predominant source of cytokines in the central nervous system and release a series of proinflammatory cytokines and chemokines, such as monocyte chemoattractant protein-1, interleukins, macrophage colony-stimulating factor, tumor necrosis factor (TNF)-α, and macrophage inflammatory protein-1α/β. Astrocytes express receptors for interleukin (IL)-1, IL-8, IL-6, and macrophage colony-stimulating factor (Kanthasamy et al., 2019). Overexpression of these pro-inflammatory cytokines and chemokines can cause neuronal damage (Chen et al., 2018; Takahashi et al., 2008). Repeated sevoflurane exposure in neonatal mice promotes activation of microglia and release of pro-inflammatory factors and increases neuroinflammatory factor (TNF‑α, IL-8, IL‑6, and IL‑1β) expression levels (Shen et al., 2013; Xia et al., 2017; Yang et al., 2020b). The PI3K/Akt/mTORpathway in the cortex and hippocampus of rats may be involved in sevoflurane-induced developmental neurotoxicity (Wang and Wang, 2019). Meanwhile, exposure of monkeys to sevoflurane during rapid brain development also promotes microglia activation, which can be detected by upregulating translocator protein (TSPO) expression (Zhang et al., 2016). Maternal exposure to sevoflurane directly influences fetal glial cells and enhances IL-6 via phospho-ERK signaling (Hirotsu et al., 2019). Neuroinflammation is involved in sevoflurane-induced developmental neurotoxicity and could be an important target for further studies of sevoflurane-induced developmental neurotoxicity.

## Assembly and plasticity of neural circuits

### Impaired synaptic plasticity directly leads to abnormal neural circuitry in early brain development

Impaired synaptic plasticity contributes to cognitive deficits, emotional disorders, and poor movement and flexibility. Brain plasticity refers to the ability of neural activity generated by experience, which can change the function of neural circuits, thereby changing the subsequent feelings, thoughts, and behaviors. Synaptic plasticity refers to the activity-dependent modification of the synaptic transmission efficiency or strength of pre-existing synapses, and has been proposed to play a central role in the brain’s ability to incorporate short-lived experiences into persistent memory traces. Synaptic plasticity also plays a key role in the early development of neural circuits, and damage to synaptic plasticity can lead to several significant neuropsychiatric disorders (Citri and Malenka, 2008). Two main types of ionotropic glutamate receptor NMDAR and α-amino-3-hydroxy-5-methyl-4-isoxazole propionic acid receptor (AMPAR) are involved in the postsynaptic glutamatergic synapse reaction. Defective hippocampal synaptic plasticity is closely related to defective hippocampal-dependent memory. Inhibition of expression of NMDAR and NMDAR subunit NR1 leads to defective LTP and spatial learning. Overexpression of NMDAR subunit NR2B in mice enhances LTP and enhances spatial learning (Citri and Malenka, 2008). Thus, synaptic plasticity plays a key role in hippocampal-dependent learning.

During critical periods of early postnatal development, brief sevoflurane exposure can induce subtle changes in synaptic plasticity and spine density (Qiu et al., 2016). Neonatal sevoflurane exposure leads to cyclin-dependent kinase 5 (CDK5) activation by increasing p25 expression (Liu et al., 2017b) and activates the CDK5/collapsin response mediator protein-2 (CRMP2) pathway and GSK-3β/CRMP2 pathways (Liao et al., 2021) in the hippocampus of neonatal rats. Exposure also suppresses cortical and hippocampal dendritic branching and reduces dendritic branch length as well as the density of dendritic spines in pyramidal neurons (Liao et al., 2021).

Additionally, exposure to sevoflurane reduces the expression of postsynaptic density 95 protein (PSD-95), synaptophysin, and drebrin in the hippocampus, which induces impaired memory in rats and inhibits LTP in hippocampal slices (Liao et al., 2021). The interaction of nectin-1 and L-afadin participates in the remodeling and formation of rat brain dendritic spines. Neonatal sevoflurane inhalation could activate corticotropin-releasing hormone (CRH)/corticotropin-releasing hormone receptor (CRHR)1 signaling to decrease nectin-1 expression levels in the hippocampus, which leads to synaptic spine loss as well as learning and memory deficits in adult mice (Li et al., 2019b). Meanwhile, sevoflurane shortens the branch length of neurons and decreases the number of branches and branch nodes (Zhang et al., 2021). Multiple sevoflurane exposures reduce synaptic function in the developing cortex (Zhou et al., 2019) and enhance HDAC6 expression and activity in the hippocampus of the developing brain, which can decrease synaptophysin and PSD-95 expression and cause synaptic ultrastructural damage and cognitive deficits in adulthood (Li et al., 2019a; Tao et al., 2016). Sevoflurane also promotes the degradation of PSD-95 protein by acting on the ubiquitinated proteasome pathway, thereby reducing PSD-95 levels (Lu et al., 2017; Wang et al., 2013a), and the expression levels of PSD-95 and synaptophysin decreased in fetus and offspring mice after pregnant mice received sevoflurane anesthesia (Zheng et al., 2013). Loss of PSD-95 releases AMPA receptors from the postsynaptic membrane, allowing subsequent removal of PSD-95 from synaptic sites by endocytosis, which leads to young mice cognitive impairment (Beique et al., 2006).

**Fig. 4:**
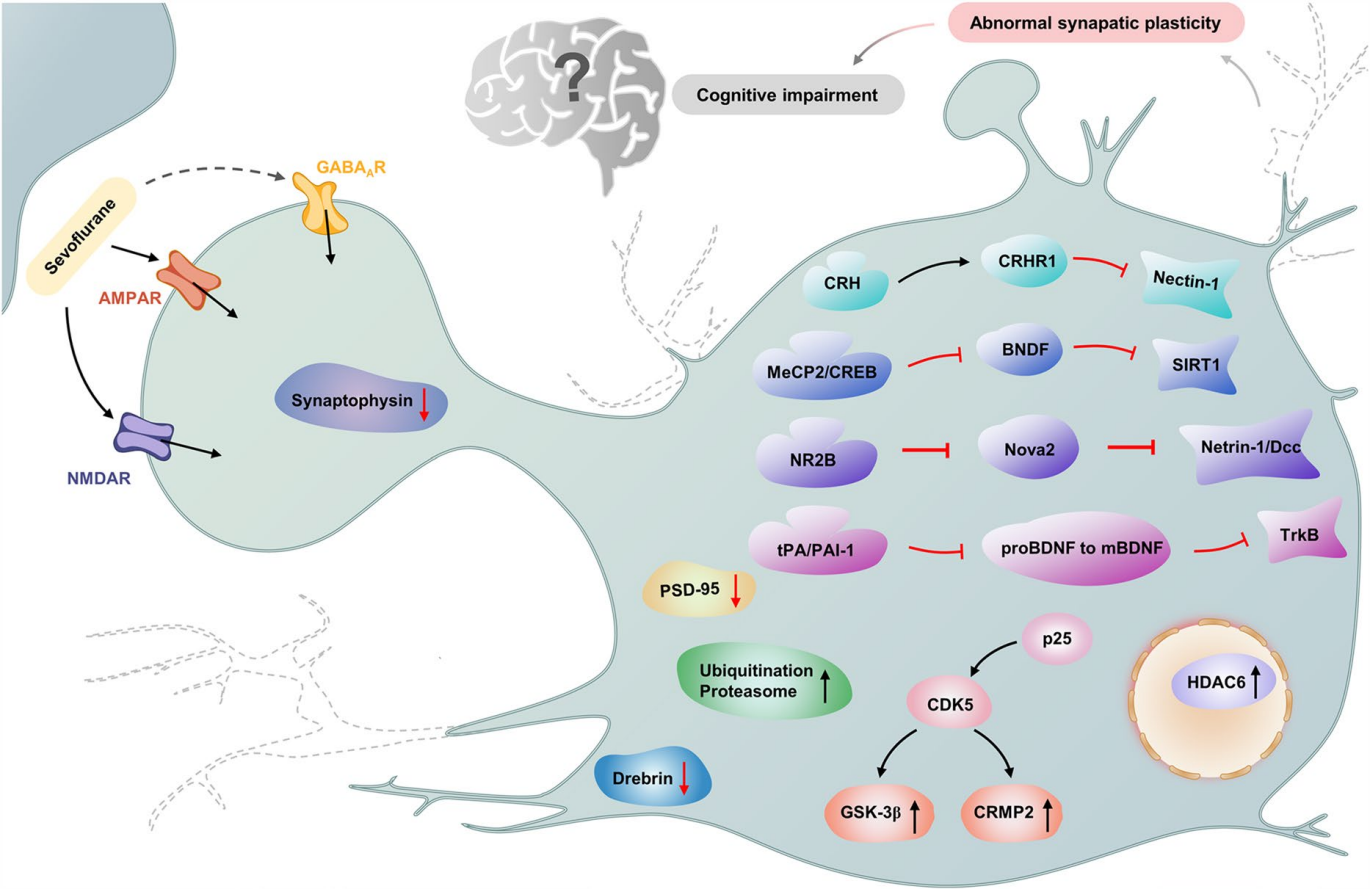
Sevoflurane regulated signaling pathways through AMPAR and NMDAR, which result in abnormal synaptic plasticity. HDAC6, P25/CDK5/GSK-3β axis, P25/CDK5/CRMP2 axis, and ubiquitination-proteasome involved in these signaling pathways, which activation could decrease PSD-95, drebrin, and synaptophysin levels, resulting in synaptic plasticity decreased and cognitive impairment. Sevoflurane could decrease synaptic plasticity through CRH/CRHR1/Nectin-1axis, MeCP2/CREB/BNDF/SIRT1 axis, NR2B/Nova2/Netrin-1/DCC axis, and tPA/PAI-1/BNDF/TrkB axis, which induce synaptic plasticity decreased and cognitive impairment also. The dotted line bids for missing synapses

Prolonged exposure to sevoflurane reduces synaptogenesis and dendritic spine formation (Yu et al., 2020b), and results in increased expression of synaptic vesicle-related proteins, decreased apical dendritic spine density, and damage to the ultrastructure of hippocampal synapses (Xiao et al., 2015), leading to cognitive functional impairments in juvenile rats. Neonatal sevoflurane exposure inhibits SIRT1 protein levels through downregulating BDNF via methylcytosine guanine phosphate-binding protein 2 (MeCP2) and CREB (Tang et al., 2020); abnormal reduction of SIRT 1 protein is associated with impaired synaptic plasticity. Sevoflurane exposure regulates the transport of NMDAR subunit NR2B and the morphology of dendritic spines (Tang et al., 2018), decreases NOVA2 expression in the developing mice cerebral cortex, inhibits Netrin-1/DCC activity in the fetal brain, interferes with the axon growth and the guidance of commissural interneurons, and reduces the migration of interneuron progenitor cells in the spinal cord (Chai et al., 2020). Multiple exposures of the developing brain to sevoflurane inhibit activation of the tyrosine kinase receptor (TrkB) signaling pathway through the imbalance of the tPA/PAI-1 fibrinolytic system and the reduction of synaptic plasticity and inhibit the cleavage of proBDNF to mBDNF (Dong et al., 2020b). Synaptic plasticity plays an important role in sevoflurane-induced neurotoxicity during development, which directly induces abnormal neural circuitry. Therefore, it is important to prevent or treat sevoflurane-associated impairment of synaptic plasticity (Fig. 4).

### Abnormal myelin development results in impaired nerve conduction

Abnormal development of myelin structures in the white matter of the brain can result in impaired nerve conduction, mainly manifested as dyskinesia and postural abnormalities, but also accompanied by sensory, cognitive, and behavioral disorders. Myelination is the process by which oligodendrocytes of the CNS or Schwann cells of the peripheral nervous system wrap axons (Choi et al., 2019; Nave and Werner, 2014), which helps provide the foundation for brain connectivity and plays an important role in cognitive development and brain plasticity (Deoni et al., 2018). In the CNS, myelination is stimulated by axonal activity and astrocytes, whereas microglia/macrophages involved in myelin clearance. Myelin sheath accelerates axon signal conduction and serves an important role in preserving the healthy connectivity and functions of nervous system (Nave and Werner, 2014). Oligodendrocytes/oligodendrocyte progenitor cells are crucial for effective myelination in the CNS (Thomason et al., 2020). Oligodendrocytes (OLs) are the myelinating cells during development and throughout adulthood in the CNS. In the process of myelination, decreased expression of OL-related genes and myelin-related genes can lead to myelin dysplasia, neuronal degeneration, and nerve injury (Ogawa et al., 2018); and OL apoptosis also leads to demyelination and neurodegeneration (Dulamea, 2017).

Sevoflurane-exposed developing nonhuman primate brains display significant apoptosis in gray and white matter, with OL apoptosis heavily concentrated in white matter zones (Ikonomidou et al., 2019; Rosado-Mendez et al., 2019), which leads to myelin dysplasia in the CNS and affects cognitive function. Exposure to high concentrations (4.9%) of sevoflurane in the early postnatal period for 2 h may have a harmful effect on the OL maturation and myelination of white matter in the brain development of rats (Wu et al., 2020b). Repeated sevoflurane anesthesia in rhesus macaques and mice leads to disrupted folate metabolism and subsequent defects in myelination in the developing brain via decreased expression of myelination-development-related genes (Zhang et al., 2019a). In the CNS, iron deficiency affects myelinogenesis, especially in the fetus or infant (Ward et al., 2014). During pregnancy, sevoflurane anesthesia in mice may inhibit myelinization in offspring via iron deficiency, leading to decreased growth of OLs, destroyed myelin integrity, reduced g-ratio of myelin sheath, and suppressed myelinization (Zuo et al., 2020). OL apoptosis, iron deficiency, and folate deficiency may be possible mechanisms of the abnormal myelin development in the developing brain caused by sevoflurane, and inhibition of OL apoptosis and supplementation of iron and folic acid may be preventive measures. More studies are needed.

### miRNAs and lncRNAs are associated with sevoflurane-induced neurotoxicity

Non-coding RNAs (ncRNAs) and their related regulatory networks are increasingly involved in mediating complex neurobiological functions. miRNAs and lncRNAs have been reported to play significant roles in neural development (Rodrigues et al., 2020; Shu et al., 2019). In the mouse hippocampus, sevoflurane induces abnormal expression of 148 mRNAs and 301 lncRNAs on PD7 (Jiang et al., 2021a). These dysregulated lncRNAs/mRNAs can form a wide range of molecular networks and may participate in various functional neurological pathways in the hippocampus, leading to acute apoptosis and impaired long-term memory. Neonatal sevoflurane anesthesia can upregulate caspase 3 and Bax, decrease the levels of Bcl‐2, BDNF, and NGF, and reduce the density of the hippocampal nerve through upregulation of lncRNA, resulting in ultrastructural changes of neuron cells and neuronal apoptosis (Hu et al., 2019). LncRNA Rik-203 (Zhang et al., 2019b), the PEG13/miR-128-3p/SOX13 axis (Jiang et al., 2020), the miR-410-3p/atrophin-1 pathway (Zhang et al., 2020b), the Gm15621/Mir-133A/SOX4 axis (Zhao and Ai, 2020), and the hsa‑miR‑302e/OXR1 axis (Yang et al., 2018) are all involved in the neurotoxicity caused by repeated sevoflurane exposure in the developing brain. Sevoflurane induces increased methylation of the presynaptic marker synaptophysin at the mRNA level and enrichment by m6A (Zhang et al., 2021), which decreases the expression of synaptophysin and leads to fine motor and cognitive impairment in young mice (Zhang et al., 2021). The damage of miRNA and lncRNA circuits causes potentially reversible and irreversible changes in brain function and structure. The role of miRNAs and lncRNAs in the anesthetic neurotoxicity of the developing brain deserves more attention.

## Tau phosphorylation is an important and new mechanism of sevoflurane-induced developmental neurotoxicity

Tau phosphorylation gives us a new perspective on sevoflurane-induced cognitive dysfunction in neonatal mice. Tau protein, as a microtubule-associated protein, first reported in 1975 (Weingarten et al., 1975). Tau predominantly functions to promote assembly and stability of microtubules, which are depressed by excessive phosphorylation of tau (Pirscoveanu et al., 2017). Excessive tau phosphorylation promotes formation of insoluble tau aggregates. Once the aggregate is formed, it can escape the original cell, contact the connected cell, enter the cell, and induce further aggregation through the template conformational change (Holmes and Diamond, 2014). These conformational changes are thought to mediate neuronal dysfunction and cognitive impairment in Alzheimer’s disease and other tauopathies. Tauopathy, including tau phosphorylation, is a hallmark of Alzheimer’s disease neuropathogenesis (Bejanin et al., 2017). Tau phosphorylation can lead to cognitive dysfunction in mice (Faraco et al., 2019). Multiple, but not single, postnatal 6-day mice exposure to 3% sevoflurane 2 h daily induced phosphorylation of tau via GSK-3β activation, which increased the level of IL-6 and decreased the level of PSD-95 in the hippocampus, leading to cognitive impairment (Tao et al., 2014). These effects of sevoflurane did not occur in tau KO mice, suggesting the contribution of tau in sevoflurane-induced neuroinflammation and synaptic deficits in mice. Sevoflurane-induced tau phosphorylation may also explain the age-dependent changes in anesthesia neurotoxicity in mice. Neonatal mice have lower levels of mitochondrial function, which causes lower ATP and higher NUAK1 amounts in the brain. Increased NUAK1 could induce tau phosphorylation at serine 356, blocking tau degradation (Lasagna-Reeves et al., 2016) and resulting in increased accumulation of tau (Yu et al., 2020a) in the brain tissues of neonatal mice compared to adult mice. Thus, neonatal mice are more vulnerable to the development of tau phosphorylation following sevoflurane anesthesia compared to adult mice.

**Fig. 5:**
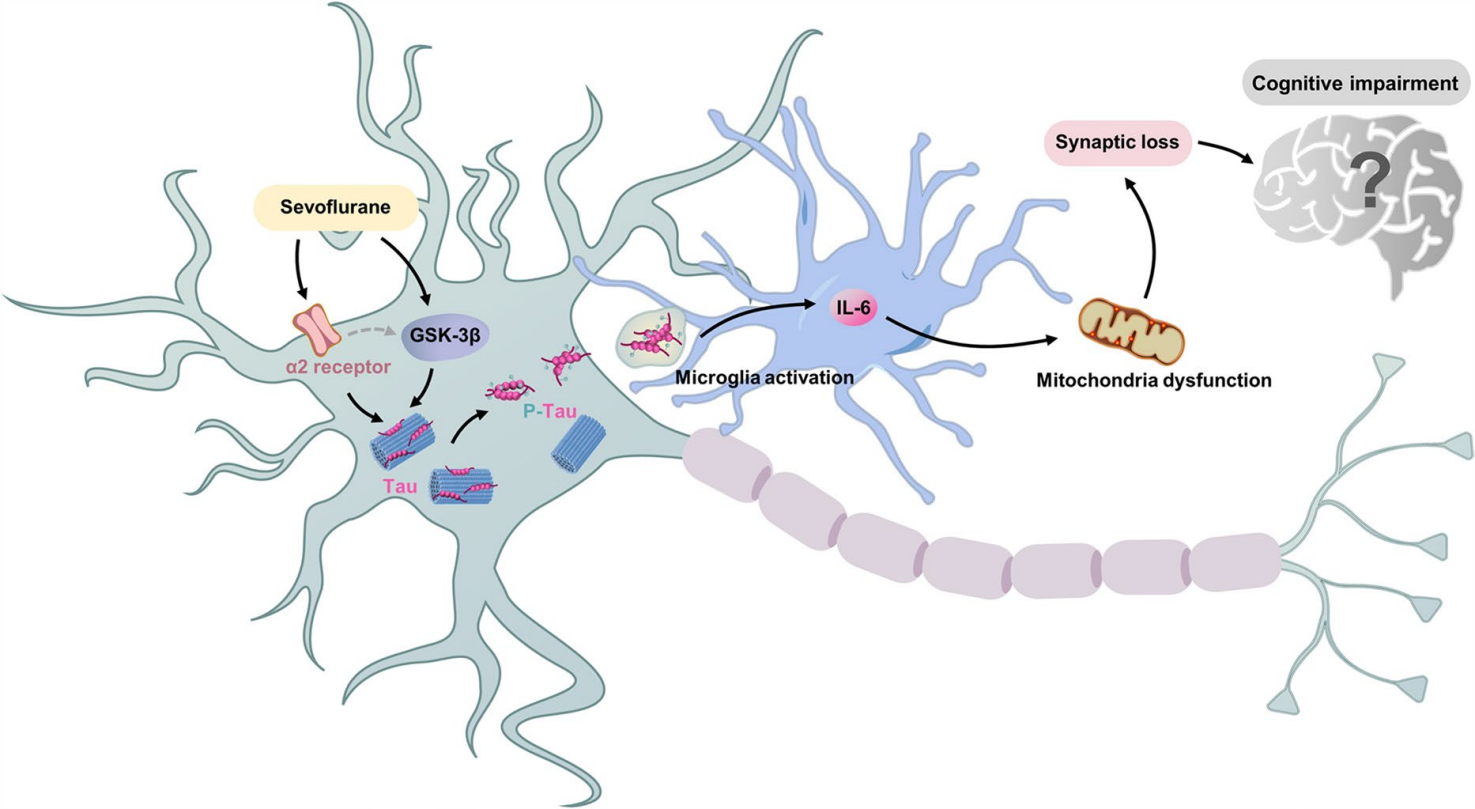
Sevoflurane increased Tau phosphorylation in the young mice through GSK-3β and/or α-2 adrenergic receptor. Tau phosphorylation could promote extracellular vesicle-associated tau trafficking from neurons to microglia, which led to microglia activation and promoted generation of IL-6.The elevation of IL-6 amounts led to mitochondria dysfunction and synaptic loss, which induced cognitive impairment

α-2 adrenergic receptor agonists dexmedetomidine can attenuate isoflurane-induced neurocognitive impairment in neonatal rats (Sanders et al., 2009). Recently, we found that α-2 adrenergic receptor agonists, dexmedetomidine and clonidine, can alleviate tau phosphorylation and cognitive dysfunction in neonatal mice induced by sevoflurane (Sun et al., 2021). Importantly, these effects can be inhibited by α-2 adrenergic receptor antagonist yohimbine (Sun et al., 2021). These data suggest that α-2 adrenergic receptor is involved in sevoflurane-induced tau phosphorylation. However, the underlying mechanism by which the α-2 adrenergic receptor contributes to tau phosphorylation remains unknown at present and deserves further study. Another recent study demonstrated that sevoflurane can induce tau phosphorylation and extracellular vesicle-associated tau trafficking from neurons to microglia, leading to generation of IL-6 and cognitive dysfunction (Dong et al., 2021). These findings suggest that we may use sevoflurane as a research tool to investigate tau trafficking and other tauopathies in vitro and in mice. Tau phosphorylation has long been shown to occur in the brain tissues of Alzheimer’s disease patients, Alzheimer’s disease transgenic mice, and aged mice. However, these new findings show that tau phosphorylation may also contribute to neurotoxicity in young brains. Future studies to further reveal the role of tau in anesthesia developmental neurotoxicity are certainly warranted (Fig. 5).

## Neuroendocrine contribution to neurotoxicity induced by sevoflurane

The neuroendocrine system may be involved in sevoflurane-induced neurotoxicity through γ-aminobutyric acid (GABA) receptors. As an important part of the neuroendocrine system, the limbic-hypothalamo-pituitary-adrenal (LHPA) axis plays an important role in the development of the nervous system and also has an important impact on learning, memory, and cognition (Hankin et al., 2015). Cl^−^ is the main charge carrier through GABA_A_R channels (Salmon et al., 2020), which is mainly regulated by Cl^−^ transporters Na + -K + -2Cl- (NKCC1) and K + -2Cl-(KCC2). Interfering with the balance of NKCC1/KCC2 may cause excessive excitement of the circuit and lead to neurodevelopmental disorders. NKCC1 and KCC2 are sensitive to neuronal damage, and the imbalance of their expression is thought to cause a variety of neuropathic diseases (Cabrera et al., 2019). Sevoflurane exposure enhances GABA_A_R activity in immature neurons, which induces increasing GABA_A_R-mediated depolarization and corticosteroid levels and electroencephalography-detectable seizures (Xu et al., 2015). 17b-estradiol contributes to the activity of GABA_A_R (Li et al., 2020), which can reduce the expression of KCC2, increase the ratio of NKCC1/KCC2 (Chastain-Potts et al., 2020; Martynyuk et al., 2020), and induce neurodevelopmental impairments. Sevoflurane induces cognitive impairment through increasing the ratio of NKCC1/KCC2 by activating GAB_A_R and increasing the level of corticosteroid, especially 17b-estradiol. These results may illustrate another mechanism by which sevoflurane causes developmental anesthesia neurotoxicity.

## Our perspective

Although there are many mechanisms by which sevoflurane induces developmental neurotoxicity, we believe that tau phosphorylation deserves more attention in the future. There are many new techniques and methods in other fields, such as single-cell omics (Wu et al., 2021), proteomics (Qiao and Wang, 2019), metabolomics (Zhang et al., 2020a), nanotechnology (Liang et al., 2016), and ultrasound/photoacoustic imaging (Li et al., 2021). These advanced technologies should be applied in future studies to paint a dynamic, multi-level, multi-dimensional picture of the molecular mechanisms of sevoflurane-induced developmental neurotoxicity. The outcomes of these studies could lead to better outcomes in caring for children.

## Conclusion

The developing brain may be uniquely vulnerable to anesthesia, pending further investigation. The mechanisms of sevoflurane-induced developmental neurotoxicity could include neural cell death, neural cell damage, impaired assembly and plasticity of neural circuits, tau phosphorylation, and neuroendocrine system abnormalities, among others. More research is needed to further reveal the underlying mechanisms by which sevoflurane and other anesthetics can induce developmental neurotoxicity.

## Data Availability

Data sharing not applicable to this article as no datasets were generated or analyzed during the current study.
